# Clinical Updates in Coronary Artery Disease: A Comprehensive Review

**DOI:** 10.3390/jcm13164600

**Published:** 2024-08-06

**Authors:** Andrea Bottardi, Guy F. A. Prado, Mattia Lunardi, Simone Fezzi, Gabriele Pesarini, Domenico Tavella, Roberto Scarsini, Flavio Ribichini

**Affiliations:** 1 Division of Cardiology, Cardio-Thoracic Department, University of Verona, 37100 Verona, Italy; bottardiandrea@gmail.com (A.B.); gfaprado@gmail.com (G.F.A.P.); simone.fezzi@aovr.veneto.it (S.F.); gabriele.pesarini@aovr.veneto.it (G.P.); domenico.tavella@aovr.veneto.it (D.T.); roberto.scarsini@univr.it (R.S.); flavio.ribichini@univr.it (F.R.); 2 Department of Clinical and Molecular Medicine, Sapienza University, 00185 Rome, Italy; 3 Department of Cardiovascular Sciences, Fondazione Policlinico Universitario A. Gemelli IRCCS, 00168 Rome, Italy

**Keywords:** coronary artery disease, dual antiplatelet therapy, percutaneous coronary intervention, coronary artery bypass graft, coronary computed tomography angiography, fraction flow reserve

## Abstract

Despite significant goals achieved in diagnosis and treatment in recent decades, coronary artery disease (CAD) remains a high mortality entity and continues to pose substantial challenges to healthcare systems globally. After the latest guidelines, novel data have emerged and have not been yet considered for routine practice. The scope of this review is to go beyond the guidelines, providing insights into the most recent clinical updates in CAD, focusing on non-invasive diagnostic techniques, risk stratification, medical management and interventional therapies in the acute and stable scenarios. Highlighting and synthesizing the latest developments in these areas, this review aims to contribute to the understanding and management of CAD helping healthcare providers worldwide.

## 1. Introduction

Coronary artery disease (CAD) is a complex pathology involving different mechanisms in its development and progression is a dynamic process that manifests itself in acute and chronic syndromes. Acute coronary syndromes (ACS) include unstable angina (UA), non–ST-segment elevation myocardial infarction (NSTEMI), and ST-segment elevation myocardial infarction (STEMI). All these syndromes involve acute coronary ischemia and are distinguished based on symptoms, ECG findings, and cardiac biomarker levels. Chronic coronary syndromes (CCS), in the past recognized as stable CAD, is a clinical entity characterized by a chronic or repetitive mismatch between supply and demand of myocardial oxygen. This includes patients with suspected CAD and either “stable” anginal symptoms/dyspnea, or with new onset of heart failure (HF) or left ventricular (LV) dysfunction, and patients with stabilized symptoms < 1 year after an ACS or recent revascularization. However, CCS includes patients with angina and suspected vasospastic or microvascular disease and asymptomatic subjects in whom CAD is detected at screening.

Although considerable progress has been made in the diagnosis and treatment of CAD in recent decades, it remains the primary cause of death worldwide. ACS are often the first clinical manifestation of CAD and in 2019 there were an estimated 5.8 million new cases of ischemic heart disease in the 57 ESC member countries. In the United States, CAD is estimated to affect 16.8 million people of these, 9.8 million have angina pectoris, and nearly 8 million have had a myocardial infarction (MI) [1]. Overall, CAD is responsible for 20% of deaths in the developed world. Consequently, substantial costs are associated with the clinical assessment of affected patients and, therefore, healthcare systems and providers face significant challenges in managing CAD. In this sense, promoting a rational allocation of resources is utmost importance, which must be based on robust scientific evidence, thereby avoiding unnecessary diagnostic examinations and medical or interventional therapies, and ultimately improving patient outcomes.

Risk stratification for CAD plays a pivotal role in determining the appropriate treatment approach, for both acute and chronic scenarios. Different strategies, both functional and anatomical as well as invasive and non-invasive, can be employed for this purpose. The evidence unequivocally supports the fact that revascularization of the culprit lesion in acute coronary syndromes leads to improved survival rates. However, emerging evidence suggests that patients in chronic scenarios may tolerate myocardial ischemia even without revascularization treatment, except in cases of severe left main disease. Consequently, this raises concerns regarding the optimal choice for assessing and treating patients with chronic coronary syndromes.

New medications and interventional procedures have emerged as valuable additions to the therapeutic arsenal for patients with CAD. These include drugs aimed at alleviating angina, low-lipid therapy, and anti-thrombotic agents and regimens, which form the cornerstone of treatment to improve both symptoms and overall survival. However, it is crucial to identify the patients who would truly benefit from these therapies, thereby avoiding unnecessary polypharmacy.

This clinical update aims to present the most recent evidence regarding the rational assessment of CAD, considering the advancements made since the last publication of the European Society of Cardiology (ESC) and American Heart Association (AHA)/ American College of Cardiology (ACC) guidelines. Our focus was primarily on risk stratification, with particular attention given to non-invasive assessment methods. We also delved into revascularization strategies and explored the latest developments in medications and procedures designed to assist healthcare professionals in their decision-making process. Additionally, we discussed future perspectives on emerging treatments for CAD, providing valuable insights into the evolving landscape of this field.

## 2. Updates in CAD Diagnosis: The Role of Coronary Computed Tomography Angiography (CCTA)

### 2.1. Traditional CAD Diagnostic Tests

While urgent invasive testing is normally required for patients with acute CAD presentation, the diagnosis of stable CAD lies in multiple functional and anatomical tests (Table 1 and Table 2).

A multimodal non-invasive diagnostic approach for CAD detection is recommended after a global clinical risk assessment. This may include anatomical (coronary computed tomography angiography (CCTA)) or non-invasive functional imaging (stress echocardiography (SE), cardiac magnetic resonance (CMR), stress-CMR, nuclear imaging, stress CCTA, or CT-derived fractional flow reserve (FFR-CT)).

#### 2.1.1. Cardiac Magnetic Resonance (CMR)

CMR is a non-invasive imaging modality to assess both the morphology, the volume, the wall motion and the function of left ventricle, and to evaluate stress perfusion defects with gadolinium-based contrast medium (GBCM) injection and the extent of scar with gadolinium enhancement (LGE) sequences.

Stress-CMR, performed after the injection of a vasodilator substance, offers a global assessment of myocardial ischemia and myocardial viability in a single examination and may be considered superior over single photon emission computed tomography (SPECT) imaging for the work-up of selected patients with known or suspected CAD [2,3,4]. In a meta-analysis by Li M. et al. stress-CMR has also been validated against FFR [5].

Furthermore, stress-CMR had comparable sensitivity and specificity to CCTA and Positron emission tomography (PET) and is superior to SPECT when using FFR as the reference standard [6].

Furthermore, a patient’s prognosis could be predicted with stress-CMR through the assessment of the infarct size, which seems to be the best predictor of mortality and significant cardiac events and through the presence of transmural necrosis which has been correlated to cardiac resynchronization therapy response and arrhythmic risk [7,8,9,10,11]. Even CMR-derived coronary flow reserve (CFR) has a prognostic role [12].

Coronary magnetic resonance angiography (CMRA) allows the non-invasive anatomical assessment of coronary arteries. Actually, clinical indications are limited to the detection of aberrant origin of coronary arteries, coronary ectasia and/or aneurysms and evaluation of bypass grafts [13,14]. A recent multi-center study showed that CMRA at 1.5 T detects significant CAD with a sensitivity of 88% and specificity of 72% and a negative predictive value of 88% [15]. Furthermore, in a direct comparison between CMRA and CCTA, no significant difference between coronary imaging at 3.0 T and 64-slice CTCA for the detection of CAD were shown [16].

CMR is one of the most promising diagnostic methods and new technologies, and improvements are moving forward. Blood-oxygen-level-dependent (BOLD) CMR uses the paramagnetic properties of deoxyhemoglobin as an endogenous contrast agent with increased deoxyhemoglobin content leading to a signal reduction on T2-weighted images reflecting myocardial oxygenation status [17,18].

#### 2.1.2. Nuclear Medicine: Single-Photon-Emission Computed Tomography (SPECT) and Positron Emission Tomography (PET)

Myocardial perfusion imaging with SPECT is a widely established approach for non-invasive assessment of patients with suspected CAD and for risk stratification.

Ischemia can be provoked by exercise, pharmacological stressors (dobutamine) or vasodilators in patients who are not able to achieve ≥ 85% of maximal age-predicted heart rate during exercise [19]. Intravenous radiotracers such as technetium-99m (99mTc) (sestamibi and tetrofosmin) and thallium-201 (201Tl) are absorbed by cardiomyocytes in proportion to myocardial blood flow and defects in tracer uptake represent either abnormal regional flow reserve or myocardial scar reflecting CAD presence [20].

PET assesses both perfusion and metabolism function with the administration of glucose analogue tracer (18F-Fluorodeoxyglucose [FDG]) and a tracer such as nitrogen 13-ammonia or rubidium-82, which remains in the vascular space, showing the distribution of myocardial blood flow. The diagnostic accuracy of myocardial perfusion by PET in the assessment of CAD has been reported to be superior to SPECT with the advantage of directly measuring and quantifying the myocardial perfusion in all left ventricular tissue. This allows the identification of ischemia even in the case of multivessel disease as well as the assessment of microvascular dysfunction [21].

Hybrid PET/CTTA imaging has enabled simultaneous integration of the coronary anatomy with myocardial perfusion and metabolism and has improved characterization of dysfunctional areas in chronic CAD increasing the diagnostic accuracy compared with PET.

#### 2.1.3. The Role of Stress Echocardiography (SE)

SE performed using either exercise or pharmacologic stressors such as dobutamine or vasodilator drugs, detects myocardial ischemia observing the transient changes in regional function. Reduced blood flow in the presence of critical epicardial coronary stenosis causes decreased wall thickening and endocardial excursion in the ischemic areas.

Although exercise, dobutamine and vasodilators have comparable effectiveness in inducing wall abnormalities in clinical practice, pharmacological SE is often preferred [22].

To improve the specificity of SE assessment, vasodilators permit incorporation of the measurements of coronary flow velocity reserve in the left anterior descending coronary artery (CFVR-LAD) [23]. Moreover, speckle-tracking echocardiography (STE) offers additional information regarding longitudinal myocardial shortening and has been shown to be superior to the visually assessed regional wall motion abnormalities.

Overall, SE demonstrates very high specificity compared to other functional tests for severe obstructive CAD detection and a high positive predictive value also in the low-risk population.

### 2.2. Risk Stratification in CAD Patients

The diagnostic process is a key point in patients with suspected obstructive CAD to identify the best management.

**Figure 1:**
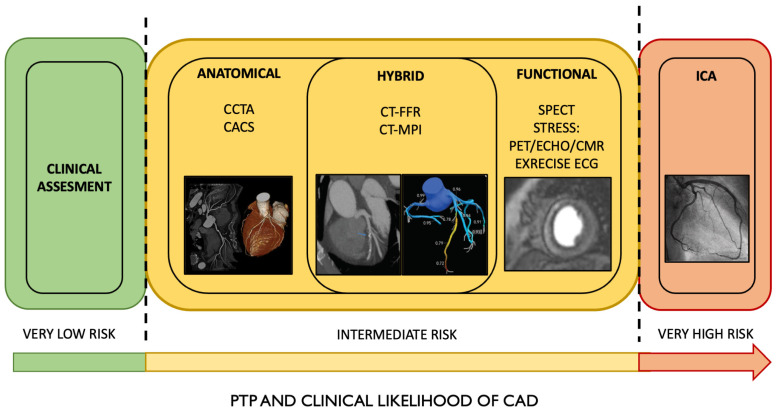
Appropriate test selection based on patient’s risk. Abbreviations: CCTA, coronary computed tomography angiography; CACS, coronary artery calcium score; CT-FFR, computed tomography-fractional flow reserve; CT-MPI, computed tomography-myocardial perfusion imaging; SPECT, single-photon emission computed tomography; PET, positron emission tomography; CMR, cardiac magnetic resonance; ICA, invasive coronary angiography; PTP, pre-test probability; CAD, coronary artery disease.

The right assessment of pre-test probability and clinical likelihood of CAD permits the selection of the most appropriate diagnostic test according to the different clinical scenarios (Figure 1).

For patients at the extreme ends of the probability spectrum, it is reasonable to rely solely on clinical evaluation to determine whether the patient does or does not have obstructive CAD without additional diagnostic testing. The effectiveness of current diagnostic methods for obstructive CAD depends on the pre-test probability of CAD, being most beneficial when this likelihood is intermediate.

The pre-test probability (PTP) of obstructive CAD is a straightforward predictive model based on age, sex, and symptoms. More comprehensive clinical models that include risk factors for cardiovascular (CV) disease (such as family history of CAD, dyslipidemia, diabetes mellitus (DM), hypertension, smoking, and other lifestyle factors), resting ECG changes, LV dysfunction indicative of ischemia, and exercise ECG findings, along with coronary artery calcium (CAC) data from CCTA, have enhanced the identification of patients with obstructive CAD compared to simpler scoring methods [24].

Framingham Risk Score (FRS) is one of the most commonly used risk algorithms, evaluating six coronary risk factors: age, gender, total cholesterol, high-density lipoprotein cholesterol (HDL), smoking habits, and systolic blood pressure [25]. The Systematic Coronary Risk Evaluation 2 (SCORE2) and SCORE2–Older Persons (SCORE2-OP) models are tailored for individuals aged 40–69 and 70–89 years, respectively, incorporating age-specific, sex-specific, and region-specific risk estimates for systolic blood pressure (SBP), smoking status, and cholesterol levels [26,27]. The Second Manifestations of Arterial Disease 2 (SMART2) model, intended for individuals with a previous CVD event, also considers chronic kidney disease (CKD) and high-sensitivity C-reactive protein (CRP) levels to predict future events in addition to the standard factors [28].

While traditional risk factors are crucial for identifying individuals at high risk of major atherosclerotic cardiovascular disease (ASCVD) events, new approaches have been developed to better characterize and monitor these individuals and those with subclinical atherosclerosis. In a recent study by Fotios Barkas et al., the integration of genetic factors (monogenic risk factors and polygenic predisposition to ASCVD), imaging techniques (CAC, carotid magnetic resonance, and femoral ultrasound [US]), and biomarkers (Apolipoprotein-B (ApoB), Lipoprotein(a) (Lpa), Interleukin-6 (IL-6), and high-sensitivity CRP (HsCRP)) showed great promise in optimizing primary CV risk assessment and management for seemingly healthy individuals with or without subclinical atherosclerosis. Additionally, future use of artificial intelligence (AI) will allow machine-learning algorithms and predictive models to identify patterns and risk factors that are not easily noticeable to physicians, enabling early detection and treatment of subclinical atherosclerosis [29].

### 2.3. Time for CCTA

CCTA stands as a powerful tool for screening patients suspected of having coronary artery disease (CAD) and for plaining treatments, particularly with recent advancements in hemodynamic functionality, including FFR-CT [30]. It has become a well-established method for evaluating patients presenting with both acute and chronic coronary syndromes [31,32]. Additionally, CCTA aids in identifying patients with non-obstructive CAD (defined as <50% stenosis), who are recognized to carry an increased risk of MI, thus allowing for the initiation of preventive therapies [33]. Furthermore, CCTA provides qualitative insights into atherosclerotic plaque composition and assists in identifying high-risk features, including positive remodeling and low-attenuation plaque [34,35]. Moreover, when obstructive CAD is excluded, consideration should be given to evaluating functional coronary microvascular dysfunction, which may contribute to angina symptoms observed in clinical entities such as MI with non-obstructive coronary arteries (MINOCA) and angina with non-obstructive CAD (ANOCA) [36]. Notwithstanding the higher number of diagnosed cases and the related increasing interest, MINOCA and INOCA remain a complex entities with different underlying atherosclerotic and non-atherosclerotic mechanisms. Among them, coronary microvascular dysfunction (CMD) represents the leading pathophysiological factor.

CMD consists of structural [37,38,39,40,41,42] and functional alterations of microcirculation [37,39,40,43], which hamper the cross-talk between coronary circulation and myocardial metabolism. Diagnostic methods include both invasive and non-invasive procedures. In the Coronary Vasomotor Disorders International Study (COVADIS), international standardized diagnostic criteria were proposed, requiring both coronary flow reserve (CFR) and index of microvascular resistance (IMR) to be measured [44].

However, to date, a non-invasive evaluation of microcirculation is feasible throughout PET, transthoracic color doppler cardiac ultrasound, perfusion CMR, and perfusion CCTA.

Therefore, CCTA might represent an appealing tool to examine not only epicardial stenosis, but also microcirculation when combinate with CCTA perfusion.

The expanding data accumulated over the past 20 years from randomized and observational studies prompted the AHA, the ACC and subspecialty societies to publish a guideline for the evaluation and diagnosis of chest pain [31]. CCTA has garnered multiple recommendations as class 1 or level A evidence, as highlighted and summarized in Table 3 [45]. Additionally, the 2023 ESC guidelines for ACS have reinforced the use of CCTA in patients with suspected ACS, non-elevated (or uncertain) high sensitive cardiac troponin (hs-cTn) levels, no ECG changes, and no recurrence of pain, incorporating CCTA or a non-invasive stress imaging test as part of the initial workup [46]. It is of note that CCTA does not have a role in patient presenting with suspicion of ongoing acute coronary occlusion, for whom emergency coronarography is the mandatory. Indeed, the RAPID-CTCA (Rapid Assessment of Potential Ischemic Heart Disease with CTCA) study demonstrated no improvement in clinical outcomes at 1 year with an early CCTA strategy and only modest reductions in the duration and cost of hospital stays, in these patients [47]. These findings also led the 2023 ESC guideline for ACS to assign a class III recommendation for routine assessment in this context. However, a significant benefit of CCTA in ACS lies in its capability to assess life-threatening differential diagnoses such as aortic dissection and pulmonary embolism. Nevertheless, the decision to extend the scan for full coverage of the aorta, neck, and pulmonary vessels should be based on the clinical context rather than following a rigid “triple rollout strategy”.

### 2.4. Coronary Artery Calcium (CAC) Scoring

An important consideration arises for individuals still stratified at low risk of obstructive CAD, specifically those falling within the 5–15% range. It has been demonstrated that incorporating information on CV risk factors, CAC scoring, or ECG changes can significantly improve the sensitivity to detect obstructive CAD compared to the traditional model, thereby facilitating physicians in prescribing further diagnostic exams [48].

CAC scoring has long been recognized as a valuable tool for stratifying patients based on their risk of future coronary events and aiding clinical decision-making regarding subsequent patient management [49]. Recently, a classification system based on CAC has been proposed and is known as the Coronary Artery Calcium Data and Reporting System (CAC-DRS). The CAC-DRS categorizes CAC based on Agatston scores into four groups: 0 (score 0), 1 (1–99), 2 (100–299), and 3 (≥300) [50]. The absence of CAC (Agatston = 0) is associated with a favorable prognosis, with an annual rate of death or MI of < 1% and a prevalence of obstructive CAD of <5% [51]. Previous study has shown that patients classified in group 3 according to the CAC-DRS classification are at significantly increased risk MI compared to those in group 0 (hazard ratio [HR] 9.41; 95% confidence interval (CI), 3.24 to 27.31; *p* < 0.001) [52]. However, it is important to note that CAC imaging does not definitively exclude the presence of non-calcified obstructive CAD, which can also contribute to adverse coronary events and the presence of CAC is a week predictor of obstructive CAD [48,51].

### 2.5. Functional Tests and CCTA

Ensuring the exclusion of patients with significant hemodynamic coronary stenosis is crucial for minimizing the need for unnecessary invasive coronary angiography following CCTA. Employing diagnostic strategies that augment the accuracy of CCTA alone holds paramount importance in this regard. Incorporating functional information for patients whose stenosis severity remains unclear following CCTA was associated with lower rates of adverse CV outcomes (death and non-fatal MI), and it is endorsed by current guidelines [30,53]. FFR-CT is derived from traditional CCTA images, which provide a detailed 3-dimensional anatomical coronary tree and physiological models. It operates on the principle of computed flow dynamics within vessels, allowing for the determination of pressure drops at every point along the coronary vessels. This approach offers distinct advantages over other non-invasive functional tests, such as positron emission tomography (PET) or single-photon-emission computed tomography (SPECT), as it obviates the need for additional examinations, medications, contrast dye, or radiation exposure [54]. Furthermore, FFR-CT analysis can be conducted offline following CCTA, thereby streamlining the diagnostic workflow. Growing evidence supports the clinical utility of FFR-CT on the setting of chronic coronary syndromes [55,56,57]. Meta-analysis comprising five studies involving 5460 patients demonstrated a significant benefit in the primary endpoint, encompassing all-cause mortality and MI at 12 months of follow-up, for patients with FFR-CT ≤ 0.80. The relative risk (RR) was 2.31 (95% CI, 1.29 to 4.1; *p* = 0.005). Additionally, each 0.10-unit reduction in FFR-CT was associated with a higher risk of the primary endpoint (RR 1.67; 95% CI 1.47 to 1.87; *p* < 0.001) [58]. The 2019 ESC guidelines for chronic coronary syndromes recommend class I functional imaging for myocardial ischemia when CCTA indicates CAD of uncertain functional significance or when the diagnosis is inconclusive. Moreover, the recent AHA/ACC/American Society of Echocardiography (ASE)/American College of Chest Physicians (CHEST)/Society for Academy Emergency Medicine (SAEM)/Society of Cardiovascular Computed Tomography (SCCT)/Society for Cardiovascular Magnetic Resonance (SCMR) guideline for the evaluation and diagnosis of chest pain classifies FFR-CT as a class IIa recommendation for intermediate-high risk patients presenting with acute or stable chest pain and exhibiting coronary stenosis of 40% to 90% in a proximal or middle coronary segment on CCTA. FFR-CT can be useful for diagnosis of vessel-specific ischemia and to guide decision-making regarding the use of coronary revascularization (Table 3).

### 2.6. CCTA for Planning Percutaneous Coronary Intervention (PCI) and Coronary Artery Bypass Graft (CABG)

Further exploration of CCTA utility has garnered attention for its potential in facilitating the planning of coronary procedures. However, despite its promise, widespread clinical utilization in daily practice remains limited. A notable study demonstrated the feasibility of real-time integration of 3D CCTA and fluoroscopic images in the catheterization laboratory to enhance PCI [59]. Additionally, an intriguing trial randomized 400 patients with chronic total occlusions (CTO) to undergo PCI with or without preprocedural CCTA guidance. The group receiving CCTA guidance exhibited a significantly higher success rate compared to those without (93.5% vs. 84.0%; absolute difference 9.5%; 95% CI, 3.4 to 15.6%; *p* = 0.003). This improvement was attributed to superior vessel tracking facilitated by a better understanding of the occlusion’s anatomical morphology derived from 3D information [60]. CCTA has been used for long to identify patent grafts in CABG patients with good accuracy [61]. However, the concept for guiding the decision-making process for revascularizations without coronary angiography is new and is of utmost relevance, especially when applying FFR-CT. A previous trial demonstrated that the treatment decision for revascularization strategies (CABG and/or PCI) based on CCTA had a high agreement with the decision derived from coronary angiography in patients with triple vessel with or without left main disease [62]. These findings suggested the possibility of referring patients to open-heart surgery without the need for traditional coronary angiography in the future. However, further studies are necessary to provide safety data in terms of guiding both percutaneous and surgical revascularization procedures with CCTA alone. The ongoing FAST TRACK CABG trial will provide evidence about this topic, investigating the safety and feasibility of CCTA and FFR-CT to plan surgical strategy and treatment without invasive coronary angiography (ICA) [63].

## 3. Revascularization Strategies in Acute Coronary Syndromes (ACS)

### 3.1. Current Overview

The adequate timing and type of revascularization treatment (primary PCI (PPCI), fibrinolysis, or CABG) represent a critical step for the management of the acute phase in ACS and are game-changers for the patient’s prognosis.

The interplay between clinical presentation (e.g., hemodynamic status) and examinations (e.g., blood tests, ECG and cardiac ultrasound) has a key role in guiding the revascularization strategy.

As a result, patients admitted with STEMI require almost immediate (<120 min) reperfusion therapy (either PPCI or fibrinolysis when indicated). Similarly, NSTEMI ACS patients benefit from an early invasive strategy (within 24 h), even urgent, when presenting any very-high-risk features [46].

Currently, PCI is the gold standard procedure for treating CAD in the acute setting, particularly in STEMI cases. However, the use of fibrinolytic agents has improved the outcomes in STEMI patients when primary PCI is not feasible in due time.

On the other side, revascularization by means of CABG represents an alternative for ACS other than STEMI in presence of complex CAD, such as left main and multivessel disease (MVD), or in patients with specific comorbidities, such as diabetes.

### 3.2. PCI vs. CABG: A Complex Approach

In STEMI patients, PPCI is the gold standard treatment for culprit lesions, while CABG should be considered only when PPCI is not feasible, particularly in the presence of ongoing myocardial ischemia and a large area of jeopardized myocardium. In clinical practice, emergency CABG is unfrequently performed because of the delay to reperfusion, surgical risks, and the low impact on prognosis.

Similar considerations can be applied to NSTEMI patients at very high risk, where PCI efficacy overcomes CABG in most cases.

Differently, in the remaining ACS cases, the choice of the revascularization depends on the number of diseased vessels, the anatomical disease complexity, and the presence of comorbidities.

In a meta-analysis merging data from the BEST (Randomized Comparison of Coronary Artery Bypass Surgery and Everolimus-Eluting Stent Implantation in the Treatment of Patients with Multivessel Coronary Artery Disease), PRECOMBAT (Premier of Randomized Comparison of Bypass Surgery vs. Angioplasty Using Sirolimus-Eluting Stent in Patients with Left Main Coronary Artery Disease), and SYNTAX (Synergy between PCI with Taxus and Cardiac Surgery) trials, the authors compared the long-term outcomes in the sub-set of patients with unstable angina (UA) and NSTEMI for left main or multivessel CAD that had undergone CABG or PCI. At 5 years follow-up, the primary outcome (a composite of death from any causes, MI or stroke) was significantly lower in the CABG-group than in the PCI-group (HR 0.74; 95% CI 0.56 to 0.98; *p* = 0.036) mainly attributed to a significant reduction in the rate of MI (HR 0.50; 95% CI, 0.31 to 0.82, *p* = 0.006). However, no difference in the rate of stroke or in the rate of deaths was revealed between the two groups [64,65,66,67].

The advantage of CABG was significant prominent in patients with multivessel CAD compared to the patients with left main disease although no significant interplay between treatment effect and the extent of CAD was found. Despite that, in patients with intermediate and high SYNTAX scores, CABG guaranteed more benefits than PCI after 6-month follow-up, suggesting that SYNTAX is a helpful score for guiding decision-making regarding revascularization.

It is of note that the SYNTAX score implemented in such studies was limited to the anatomical score (first version of the score), while the use of its more recent version, which includes clinical and functional parameters and predicts prognosis up to 10 years, may even better address patients to either treatment.

In absence of specific randomized clinical trials (RCT), revascularization strategy in acute settings remains uncertain. Patients hemodynamically unstable and at high risk should be treated with PCI, while in complex cases with multivessel or left main disease—associated with comorbidities such as diabetes—heart team discussion is recommended, and CABG represents an alternative strategy that could improve long-term outcomes.

### 3.3. Complete vs. Non-Complete Revascularization and Appropriate Timing

Half of patients with ACS present MVD, which is associated with worse prognosis and lower successful reperfusion rates after PPCI [68]. The correct management of non-culprit lesions has been deeply investigated in some RCT.

The PRAMI (Preventive Angioplasty in Myocardial Infarction) trial enrolled STEMI patients with multivessel CAD (one or more stenosis ≥ 50% in non-infarcted related arteries) as detected at the time of emergency PCI, who were randomly assigned to preventive PCI of the non-culprit lesions vs. a conservative strategy. The primary outcome (a composite of death from cardiac causes, nonfatal myocardial infarction, or refractory angina) occurred less frequently in the preventive PCI group (HR 0.35; 95% CI, 0.21 to 0.58; *p* < 0.001) [69].

In the COMPLETE (Complete vs. Culprit-only Revascularization to Treat Multivessel Disease After Early PCI for STEMI) trial, STEMI patients with multivessel CAD (at least one severe non-infarction-related lesion defined angiographically or functionally with FFR value ≤ 0.80) that had undergone a successful PPCI were randomized to a complete revascularization strategy vs. conservative management of non-culprit lesions. The two coprimary endpoints (the composite of CV death or MI and the composite of CV death, MI, or ischemia-driven revascularization) were significantly inferior in the complete revascularization group (HR 0.74; 95% CI, 0.60 to 0.91; *p* = 0.004 and HR 0.51; 95% CI, 0.43 to 0.61; *p* < 0.001 respectively). Moreover, complete revascularization improved patients’ health status without increasing the rate of acute kidney injury (AKI) and major bleeding. It is of note that the timing (during index hospitalization or after hospital discharge but within 45 days) of non-culprit lesions PCI did not impact the clinical outcomes.

The clinical benefits with complete revascularization strategy emerged from 45 days to the end of follow-up, underscoring the concept that early events after STEMI are generally related to the culprit lesion. In addition, the large proportion of thin-cap fibroatheroma of non-culprit lesions, revealed with optical coherence tomography (OCT), may explain better that the benefits of full revascularization strategy increase over time [70].

Similar results were also identified in the CvLPRIT (Complete vs. Lesion-only Primary PCI Trial) trial, in the DANAMI-3-PRIMULTI (Third Danish Study of Optimal Acute Treatment of Patients with ST-Segment Elevation Myocardial Infarction—Primary PCI in Multivessel Disease) trial, and in the COMPARE-ACUTE (Comparison Between FFR Guided Revascularization vs. Conventional Strategy in Acute STEMI Patients With MVD) trial regardless of the use of physiology to guide revascularization of non-culprit lesions [71,72,73]. In the COMPARE-ACUTE trial, the reduction of primary composite endpoint (all-cause mortality, non-fatal MI, any revascularization, or cerebrovascular events at 12 months) in the FFR group was mainly driven by the lower frequency of revascularization. However, issues of the reliability of FFR measurements in the acute MI setting are still debated. The coronary microcirculation is jeopardized during an acute MI, and the value of FFR measured in that condition may differ from that in a more stable setting (smaller flow increase, yet underestimated FFR for any stenosis severity).

The data from 10 RCTs, for a total of 7030 patients presenting with STEMI and multivessel CAD, were included in a meta-analysis and corroborates a complete revascularization strategy in this setting. Accordingly, the treatment of non-culprit lesions led to a significant reduction in CV mortality, while no difference was found in all-cause mortality between the two groups [74].

Compared to STEMI, the identification of the culprit lesion in NSTEMI patients is sometimes complex, remaining uncertain in up to 35% of cases, making the investigation about the completeness of revascularization more challenging.

To date, data from meta-analysis of non-randomized studies support a complete revascularization strategy even in NSTEMI patients [75,76].

However, in this setting, no RCT has been completed investigating a culprit only or a full revascularization strategy, while some investigators are focusing on the optimal timing for non-culprit lesion revascularization.

In the SMILE (Single-Staged Compared with Multi-Staged PCI in Multivessel NSTEMI Patients) trial, NSTEMI patients with MVD were randomly assigned to 1-stage percutaneous coronary intervention (1S-PCI) during the index procedure vs. multistage percutaneous coronary intervention (MS-PCI). The incidence of major adverse cardiovascular and cerebrovascular events (MACCE) at 1 year was significantly lower in the 1S-PCI-group (HR, 0.549; 95% CI, 0.363 to 0.828; *p* = 0.004), mainly due to a significantly lower rate of target vessel revascularization (TVR). The reason for this significant difference is unclear, although a possible explanation may be the higher number of 6-month stress tests executed in MS-PCI group during the follow-up. Moreover, the rapidly decreasing of myocardial enzymes, especially Troponin T (TnT), in the 1S-PCI group is probably related to a shorter time of myocardial ischemia and the absence of erroneous identification of the culprit lesion [77].

**Figure 2:**
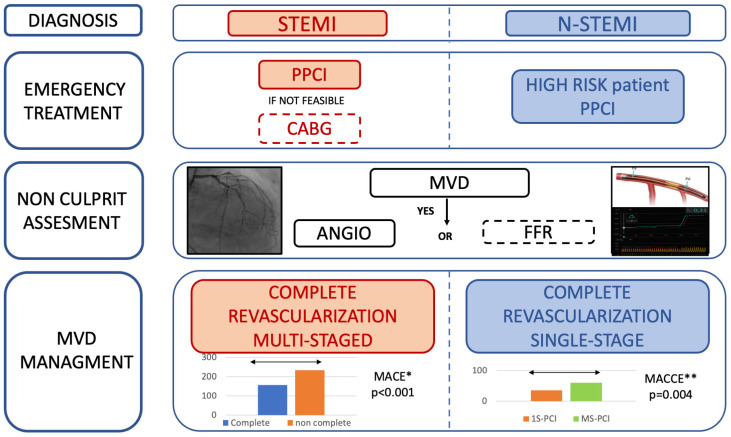
Summary of the revascularization modalities for patients presenting with MI. Abbreviations: NSTEMI, non-ST segment elevation myocardial infarction; STEMI, ST segment elevation myocardial infarction; PPCI, primary percutaneous coronary intervention; CABG, coronary artery bypass grafting; MVD, multivessel disease; FFR, fractional flow reserve. * referred to COMPLETE trial. ** referred to SMILE trial.

In conclusion, although STEMI and NSTEMI have a different pathophysiology, in both setting PCI of non-culprit lesions seems reducing mortality and recurrent MI. The optimal timing to complete revascularization has not yet been elucidated, though in NSTEMI patients, some evidence suggests a benefit in performing PCI of all significant lesions in a single procedure, especially in patients with good clinical conditions, simple coronary anatomy, and hemodynamic stability (see Figure 2).

### 3.4. Severity Assessment of Non-Culprit Lesions: Angiography or Physiology?

Considering the prognostic relevance of a complete revascularization, the assessment of non-culprit lesions severity assumes a key-role in the management of ACS patients. It is still undetermined if FFR and similar indexes are reliable in the context of ACS, where potential microvasculature alterations may affect the physiological evaluation. However, some non-RCT and RCT examples suggest its effectiveness and accuracy even during the PPCI [78,79,80].

On the other side, the FLOWER-MI (Flow Evaluation to Guide Revascularization in Multivessel ST-Elevation Myocardial Infarction) trial showed contrast results. In patients with STEMI and MVD, a FFR-guided complete revascularization did not impact significantly in reducing the risk of death from any cause, with nonfatal MI or unplanned hospitalization leading to urgent revascularization at 1-year follow-up compared to angiography-guided strategy (HR, 1.32; 95% CI, 0.78 to 2.23; *p* = 0.31) [81]. Further data from this study identified a numerically higher rate of non-fatal MI and urgent revascularization in the FFR-group, questioning the actual usefulness of physiology in such a context. Indeed, the increase in hyperemic microvascular resistance and a blunted adenosine responsiveness during the acute phase, associated with infarct size, may modify the correct assessment of the stenosis.

However, in the FAMOUS NSTEMI (Fractional Flow Reserve vs. Angiographically Guided Management to Optimise Outcomes in Unstable Coronary Syndromes) trial, NSTEMI patients with at least one coronary stenosis ≥ 30% were randomized to angiography vs. FFR assessment of non-culprit lesions. Major adverse cardiovascular events (MACE) were similar between treatment groups, although periprocedural MI tended to be higher in the angiography-guided group, and spontaneous MI tended to be higher in the FFR-guided group.

Remarkably, at 12 months, the need for revascularization was lower in the FFR-guided group, but no significant differences in health outcomes and quality of life between the groups were reported [82].

In a subgroup of the FAME (Fractional Flow Reserve vs. Angiography for Multivessel Evaluation) trial in patients with MVD and UA or NSTEMI, no difference was observed in procedural success, procedure time, or duration of hospital stay, except for the greater amount of contrast used in the angiography-guided group compared to the FFR-guided group. Moreover MACE were comparable between the two groups [83].

While according to these results, similarly to STEMI patients, physiology-guided revascularization in this setting does not seem to improve hard endpoints, the FRAME AMI trial (FFR vs. Angiography-Guided Strategy for Management of AMI With Multivessel Disease) suggests the opposite. FFR-guided decision making of non-culprit lesion reduced the rate of primary endpoint (composite of death, MI and urgent revascularization) compared to a strategy of routine PCI based on angiographic diameter stenosis in patients with STEMI or NSTEMI and MVD at 3.5-year follow-up (HR, 0.43; 95% CI, 0.25 to 0.75; *p* = 0.003). The benefit of FFR-guided PCI on the primary endpoint was consistent regardless of STEMI or non-STEMI. Moreover death and unplanned revascularization were indeed more likely after angiography guided non-culprit PCI [84].

In conclusion, in ACS patients, the FFR assessment represents a safe and useful tool to guide revascularization of non-culprit lesions, leading to fewer PCIs. However, its impact in terms of major cardiac events remains unclear, and further trials are needed to clarify such an aspect (see Figure 2).

### 3.5. Cardiogenic Shock in ACS Patients

Cardiogenic shock (CS) is the leading cause of death in patients with MI in the PCI era. The mortality could be reduced with an early revascularization, especially with PPCI. In the SHOCK (Should We Emergently Revascularize Occluded Coronaries for Cardiogenic Shock) trial, the reduction on the overall mortality was only significant beyond 6 months after the early revascularization (*p* = 0.027) while no statistical difference was found at 30-day follow-up compared to the medical therapy (*p* = 0.11) [85].

MVD is related to poor prognosis and involves a large number, up to 80%, of patients with a diagnosis of STEMI complicated by CS. In such cases, guidelines do not offer specific recommendations on the management of non-culprit lesions, though it may be reasonable to perform a complete revascularization if cardiogenic shock persists after the treatment of the culprit artery. In the Culprit Lesion Only PCI vs. Multivessel PCI in Cardiogenic Shock (CULPRIT-SHOCK), trial the PCI of the culprit lesion only reduced the rate of mortality, mainly driven by lower dose of contrast used, with less acute left ventricular volume overload and a subsequent negative effect on MI and recovery [86].

Mechanical circulatory support (MCS) in this clinical setting might render the revascularization safer as a result of the afterload reduction and the diastolic function improvement with better coronary perfusion. However, the use of intra-aortic balloon counterpulsation (IABP) before or after the start of the procedure did not impact significantly on the mortality at 30-day follow-up (RR with IABP 0.96; 95% CI, 0.79 to 1.17; *p* = 0.69) [87]. Although Impella CP may provide a more impacting hemodynamic support compared to IABP (3–3.5 L/min vs. 0.8–1 L/min) no significant mortality benefits were found at 30-day or 6-month follow-up in the IMPRESS in Severe Shock trial (Impella vs. IABP Reduces Mortality in STEMI Patients Treated With Primary PCI in Severe Cardiogenic Shock). On the contrary, vascular and bleeding complications were higher using Impella CP driven by different sheath sizes (14 Fr for Impella CP vs. 7.5 Fr for IABP) [88].

Patients with CS present high rates of mortality and challenging management. PCI should be limited to the culprit lesion during the acute emergency setting, and a complete revascularization should be evaluated after clinical stabilization. The benefits of MCS remain uncertain, and it requires more RCT to clarify its real impact on mortality.

## 4. Revascularization Strategy in Chronic Coronary Syndrome (CCS)

### 4.1. Current Overview

The role and impact of revascularization in CCS is still debated, and the right choice between invasive strategies associated with medical therapy or medical therapy alone is not straightforward in routine practice.

While the historical treatment of CCS consisted in the medical therapy alone, the advent of PCI was considered an add-on to relieve symptoms, improve quality of life (QoL), and prolong survival [89].

Consequently, PCI gained a key role in the treatment of such disease, although some studies have shown no significant impact on reducing the incidence of death and MI in spite of an improvement in angina symptoms and short-term exercise performance [90,91].

### 4.2. Revascularization or Medical Therapy?

The seven-year data of the RITA-2 (second Randomized Intervention Treatment of Angina) trial reflect the long-term effects of PCI plus medical therapy in comparison with medical therapy alone in patients with stable CAD. After seven-year follow-up, an initial strategy of PCI did not influence the risk of death or MI but improved angina and exercise tolerance [92].

Similarly, in the COURAGE (Clinical Outcomes Utilizing Revascularization and Aggressive Drug Evaluation) trial, no significance difference between the two groups occurred in the primary outcome (composite of death from any cause and nonfatal MI) as well as in the 4.6 year cumulative primary event rates (19% vs. 18.5%; unadjusted HR for the PCI group, 1.05; 95% CI, 0.87 to 1.27; *p* = 0.62) and in the prespecified composite outcome of death, nonfatal MI, and stroke (20.5% vs. 19.5%; HR, 1.05; 95% CI, 0.87 to 1.27; *p* = 0.62). The lower-than-projected event rate in the medical therapy group may depend on the type of atherosclerotic plaque morphology and vascular remodeling in the stable CAD, which makes medical therapy able to reduce plaque vulnerability. However, at 4.6 years of follow-up, medical therapy group had significant higher rates of additional PCI (HR, 0.60; 95% CI, 0.51 to 0.71; *p* < 0.001).

In both groups, there was a consistent reduction in the relief of angina during follow-up despite significantly lower values in the PCI group at 1 year (*p* < 0.001) and at 3 years (*p* = 0.02), while at 5-year follow-up, the differences between the two groups disappeared (*p* = 0.35) [93,94].

Further investigations suggested that a real prognostic benefit of PCI could derive from the use of physiology tests to guide revascularization in order to define the presence of significant myocardial ischemia [95,96].

The FAME II (The Fractional Flow Reserve vs. Angiography for Multivessel Evaluation) trial showed that in patients with angiographic evidence of CAD, FFR-guided PCI in addition to medical therapy was superior to medical therapy alone in the management of patients with stable angina. The rate of primary endpoint (composite of death, MI, or urgent revascularization) was significantly lower in the PCI group than in the medical-therapy group (4.3% vs. 12.7%; HR with PCI, 0.32; 95% CI, 0.19 to 0.53; *p* < 0.001), mainly driven by the lower rate of urgent revascularization in the PCI group (1.6% vs. 11.1%; HR, 0.13; 95% CI, 0.06 to 0.30; *p* < 0.001). Moreover, patients in the PCI group were significantly less likely to undergo any revascularization or nonurgent revascularization [97,98].

At 5 years, the primary endpoint was confirmed between the two groups (13.9% vs. 27.0%; HR, 0.46; 95% CI, 0.34 to 0.63; *p* < 0.001), as was the rate of urgent revascularization. However, there were no significant differences between the two groups in the rates of death or MI [99].

Interestingly, the relationship among FFR, angina, and improvement in health status after PCI in patients with stable CAD was evaluated in the “Fractional Flow Reserve and Quality-of-Life Improvement After Percutaneous Coronary Intervention in Patients with Stable Coronary Artery Disease” trial. A lower baseline FFR value and a higher angina class were associated with greater improvements in health status 1 month and 1 year after PCI [100].

In a recent meta-analysis pooling the results of 25 trials, patients with stable CAD that had undergone elective revascularization plus medical therapy had lower risk of cardiac death (RR 0.79, 0.67 to 0.93; *p* < 0.001) and spontaneous MI (RR 0.74, 0.64 to 0.86; *p* < 0.01) at 5.6 average years of follow-up. However, no significant difference was found between the two strategies in all-cause mortality (RR 0.94, 0.87 to 1.01; *p* = 0.11), in any MI (both procedural and spontaneous; RR 0.87, 0.73 to 1.05; *p* = 0.14), as well as in stroke risk (RR 1.18, 0.86 to 1.60, *p* = 0.30) [101].

The most recent ISCHEMIA (International Study of Comparative Health Effectiveness with Medical and Invasive Approaches) trial enrolled patients with stable CAD and moderate to severe ischemia on stress-testing (although FFR evaluation was not provided), randomly assigned (1:1) to an initial invasive strategy of medical therapy plus revascularization when feasible or to an initial conservative strategy of medical therapy alone. Among the invasive strategy group, 79% of patients underwent revascularization (PCI in 74% and CABG in 26%).

The primary outcome at 38 months follow-up (a composite of death from CV, MI, or hospitalization for UA; heart failure; or resuscitated cardiac arrest) occurred in 12.2% and in 13.5% patients, respectively, for the invasive strategies group and the conservative strategies group (HR 0.93; 95% CI, 0.80 to 1.08; *p* = 0.34).

The cumulative event rate at 6 months was 5.3% in the invasive group and 3.4% in the conservative one (difference, 1.9 percentage points; 95% CI, 0.8 to 3.0). At 5 years, the composite endpoints amounted to 16.4% and 18.2%, respectively (difference, −1.8 percentage points; 95% CI, −4.7 to 1.0). Results were similar with respect to the key secondary outcome.

The early increased risk of the primary and major secondary outcomes in the invasive-strategy group was due to a higher rate of peri-procedural MI in the early follow-up, while they had fewer spontaneous MIs during the follow-up [102].

During extended follow-up, all-cause mortality was similar between treatment groups (*p* = 0.74). However, invasive therapy was associated with a reduction in CV mortality (*p* = 0.008), that was offset by an increase in non-CV mortality (*p* = 0.016).

In the light of such findings, medical therapy, when optimally managed, remains the landmark treatment of CCD. In this context, an invasive treatment should be limited only to functionally significant lesions, or to those causing refractory angina despite anti-anginal medications.

The ISCHEMIA trial has questioned the value of percutaneous treatment in stable patients with moderate or severe ischemia as diagnosed by a stress test.

However, the trial presents some limitations. Firstly, statistical power was decreased by reducing the sample size from 8000 to 5179 patients, and event rates were lower than expected within a modest period of follow-up. Second, most patients included were asymptomatic or only mildly symptomatic at baseline. This is in contrast with one of the revascularization effects, namely the angina relief, which could not be evaluated in such cases. However, these findings suggest that medical therapy in asymptomatic or mildly symptomatic patients might be the best initial strategy, whereas invasive strategy would be a reasonable complementary approach for symptomatic patients with frequent refractory angina episodes.

Interestingly, in the more recent Preventive PCI versus Medical Therapy Alone for treatment of vulnerable Atherosclerotic Coronary Plaque (PREVENT) trial, patients with no flow limiting (FFR > 0.80) coronary stenosis but presenting features of vulnerability (defined by intracoronary imaging) were assigned to either preventive PCI plus optimal medical therapy or optimal medical therapy alone. At two years the primary outcome (composite of death from cardiac causes, target-vessel MI, ischemia-driven TVR, hospitalization for UA or progressive angina) was significantly lower in the PCI group (absolute difference −3.0%; 95% CI, −4.4 to −1.8; *p* = 0.0003). Moreover, the benefit of preventive PCI was confirmed for each component of the primary composite outcome up to 7-year follow-up [103].

These findings might change the current practice on the management of non-functionally significant lesions.

### 4.3. Revascularization Strategies: PCI vs. CABG

For decades, CABG has represented the choice of treatment of CAD thanks to the continue innovation of surgical technique and post-operative cares which allowed to reduce morbidity, mortality and the rates of graft occlusion [104,105,106,107].

The advent of PCI, initially with bare-metal stent (BMS) followed by drug-eluting stents (DES), has guaranteed a valid alternative to surgical revascularization even in presence of complex coronary lesions anatomy, and clinical risk factors.

So far, researchers have performed multiple studies to clarify the best revascularization strategy according to different clinical and anatomical scenarios.

The Synergy between PCI with Taxus and Cardiac Surgery (SYNTAX) trial was a milestone that showed superiority of CABG compared to PCI in patients with left main or three-vessel disease.

However, the rates of death and MI at 1 year were similar between patients who underwent CABG and those who underwent PCI with DES, whereas the rate of stroke was increased in the CABG group (2.2% vs. 0.6% with PCI, *p* = 0.003) and the rate of repeat revascularization was increased in the PCI group (13.5% vs. 5.9%, *p* < 0.001).

In the SYNTAX trial, most cases of stent thrombosis occurred within 30 days after the procedure, and the 12-month rate of stent thrombosis in the PCI group was similar to the rate of symptomatic graft occlusion in the CABG group. However, as described in the literature, stent thrombosis often has more serious consequences for patients (rate of death, approximately 30%; rate of MI > 60%) than graft occlusion, which often results only in angina leading to revascularization.

According to the results, the authors developed the SYNTAX score, which was predictive of outcomes in patients who underwent PCI. Indeed, the higher the SYNTAX score, the higher the rate of all MACCE. Moreover, patients with low or intermediate Syntax score (<33) had similar rates of MACCE regardless of revascularization strategy [67].

Data at 5 years of follow-up in patients with high or intermediate SYNTAX scores suggested the trend remained the same and MACCE amounted at 26.9% after CABG and at 37.3% after PCI (*p* < 0.0001) [108].

It is of note that the follow-up of the SYNTAX trial has been extended up to 10 years, aiming to collect the survival status of the patients. Remarkably, in the very long term, there was no significant difference in all-cause mortality in the PCI-group vs. the CABG-group. However, patients with three-vessel disease presented a significant survival benefit with CABG compared to those with CAD limited to left main [109].

The SYNTAX score II was developed to help heart teams in the decision-making process considering both patient’s clinical and anatomical features. The former include DM, age, sex, renal function (defined by the Cockcroft–Gault formula), peripheral vascular disease (PVD), chronic obstructive pulmonary disease (COPD), and left ventricle ejection fraction (LVEF), while anatomical factors include the angiography presence of unprotected left main disease combined with the anatomical (first version) SYNTAX score [110].

The SYNTAX II trial, validating such a score, showed that a SYNTAX II-based strategy was associated with a significantly lower rate of MACCE, including a lower rate of repeat revascularization, spontaneous MI, and lower rate of cardiac death when compared to patients’ outcomes after undergoing PCI on the basis of the anatomical SYNTAX alone [111].

More recently, a new updated version of the SYNTAX II score was released, SYNTAX score 2020. This version uses two anatomical effect modifiers (the anatomical SYNTAX score and the presence of three-vessel disease or unprotected left main disease) while adding current smoking and removing gender from the clinical/demographic factors to predict 5-year all-cause death and MACE rates after PCI or CABG. The SYNTAX score 2020 has been externally validated for 5-year outcomes using patient-level data from landmark randomized controlled trials, including FREEDOM, BEST, PRECOMBAT, and EXCEL trials [112].

Similarly to the SYNTAX trial, in the BEST (the Randomized Comparison of Coronary Artery Bypass Surgery and Everolimus-Eluting Stent Implantation in the Treatment of Patients with Multivessel Coronary Artery Disease) trial, PCI with everolimus stents failed to show non-inferiority to CABG in this setting.

However, an interesting finding, opposite to the previous trial, was the absence of difference in stroke rate between the two groups probably related to the use of off-pump CABG that permit to avoid excessive manipulation of the aorta [113].

A meta-analysis including 23 trials confirmed that PCI was associated with a higher rate of all-cause (incidence rate ratio, 1.17; 95% CI, 1.05 to 1.29) and cardiac (incidence rate ratio [IRR], 1.24; 95% CI, 1.05 to 1.45) mortality at five-year follow-up [114].

As reported in the SYNTAX population, patients with multivessel CAD and diabetes are extremely frequent [115]. This subset of patients represents a peculiar entity, often requiring dedicated studies.

Contrary to the BARI (Bypass Angioplasty Revascularization Investigation) trial and to a sub-study of the SYNTAX trial, in the Coronary Artery Revascularization in Diabetes (CARDia) trial, no significant differences occurred in the composite of death, MI, and stroke regardless of the revascularization strategy used. However, treatment with PCI in diabetic patients was associated with an increased incidence of late MI and the need for repeat revascularization at 1 year [116,117].

Different findings were showed in the FREEDOM (the Future Revascularization Evaluation in Patients with Diabetes Mellitus: Optimal Management of Multivessel Disease) trial. Indeed, patients with DM who underwent CABG had decreased incidence of MI and all-cause mortality (at 5 years) compared to those who underwent PCI. The benefit of CABG was mainly driven by the lower rates of MI (*p* < 0.001) and death from any cause (*p* = 0.049), while strokes showed the opposite trend.

However these data are not appliable at the entire population because only patients with low surgical risk were enrolled [118].

A meta-analysis of RCT comparing CABG with PCI (with either BMS or DES) in these patients showed that at 5 year CABG led to lower all-cause mortality than PCI (RR 0.67; 95% CI, 0.52 to 0.86; *p* = 0.002) [119].

According to such results, patients presenting with multivessel CAD and diabetes have a consistent prognostic benefit when treated with CABG, compared to PCI.

### 4.4. Left Main Revascularization: PCI or CABG?

Due to the large amount of subtended myocardium, left main CAD deserves particular attention. The continuous advances of medical devices, including new-generation DES and tools for intracoronary imaging, have made PCI almost equivalent to CABG in terms of efficacy and safety during left main revascularization.

In this context, a conservative management was found to be clearly inferior to revascularization since the time of the first CABG studies, showing a significantly higher 5-year mortality in comparison to CABG [120].

With the advent of PCI, researchers initially failed to demonstrate the non-inferiority of PCI compared to CABG, mainly due to the use of older-generation stents, absence of intracoronary imaging techniques, and study design inappropriate for their aim.

In the PRECOMBAT (Premier of Randomized Comparison of Bypass Surgery vs. Angioplasty Using Sirolimus-Eluting Stent in Patients with Left Main Coronary Artery Disease) trial, PCI with sirolimus-eluting stents was shown to be non-inferior to CABG in term of MACCE at 12 months (cumulative event rate, PCI 8.7% vs. CABG 6.7%; absolute risk difference 2.0%; 95%CI, 1.6 to 5.6; *p* = 0.01 for noninferiority). However, among patients that underwent surgical treatment, the rate of ischemia-driven TVR was lower than in the PCI group [121].

However, in the EXCEL (Evaluation of XIENCE vs. Coronary Artery Bypass Surgery for Effectiveness of Left Main Revascularization) trial, PCI (with everolimus-eluting stents) was non-inferior to CABG in patients with critical left main stenosis and low–intermediate SYNTAX score with respect to the primary composite outcome of death, stroke, or MI up to 3-year follow-up (difference, 0.7%; 97.5% upper CI 4.0%; *p* = 0.02 for noninferiority; HR 1.00; 95% CI, 0.79 to 1.26; *p* = 0.98 for superiority).

However, as expected, adverse clinical events were not uniformly distributed from a temporal standpoint between the two arms. Indeed, the rate of the composite end-point was higher with CABG in the first 30 days, and clinical outcomes were actually better with PCI up to 30 days. This reversed between 30 days and 3 years such that outcomes occurred less in the CABG group than in the PCI group.

Again, after 3-years follow-up, ischemia-driven revascularization was more frequent after PCI than after CABG [122,123,124].

Otherwise, the NOBLE (Nordic–Baltic–British Left Main Revascularization) trial failed to show non-inferiority, and CABG was found to be superior to PCI for the primary endpoint of MACCE [125].

In the current trial periprocedural MI was excluded from the primary endpoint, which indeed was the game-changer in the EXCEL trial.

Comparing the two trials several differences are worth of mentioning: the surgical risk of the study population was higher in the EXCEL trial, different stents were used (everolimus-eluting stents in the EXCEL, while first generation DES and biolimus eluting stent (BES) in the NOBLE), and different endpoint definitions were adopted. Despite that, results were more comparable than we may have expected. Indeed, between the two studies, no differences were shown with respect to the composite endpoint, including all-cause of death, CV death, periprocedural an non procedural MI and revascularization. Moreover, only mortality was found to be higher in the EXCEL trial, likely reflecting the higher baseline risk profile of the patients enrolled.

Differently, in the NOBLE trial, more strokes were reported in the PCI group (data not observed in any other RCTs). In addition, probably due to the first-generation DES, a higher stent thrombosis rate was recorded compared to that of an everolimus-eluting stent (EES). Indeed, external data suggest comparable outcomes between the BES and EES [73]. These data suggest stent selection may be fundamental in the context of complex PCI such as left main or bifurcations.

To date, despite the technological improvement of percutaneous techniques, CABG seems to offer more advantages in the long term, mainly due to fewer ischemia-driven revascularizations. However, on the top of such studies, the best treatment choice is left to a multidisciplinary heart team discussion.

### 4.5. Revascularization in the Specific Setting of Heart Failure with Reduced Ejection Fraction

While most trials enrolled patients with preserved LVEF, a number of patients with significant CAD also present heart failure with reduced LVEF, and the decision on how to treat coronary stenosis is currently controversial.

Previous trials have shown different survival rates according to the number of diseased vessels: patients with single-vessel or two-vessel disease have similar outcomes regardless of the type of treatment (CABG or PCI), while patients with three-vessel disease have better long-term survival if treated with CABG [126].

Some may argue that medical therapy could be more beneficial in this category of patients [127], in particular considering the remarkable efficacy of anti-ischemic and anti-remodeling molecules available today.

In the STICH (Surgical Treatment for Ischemic Heart Failure) trial, patients with ischemic left ventricle systolic dysfunction (LVEF < 35%) had a reduction in the rates of CV death at 56 months with CABG plus medical therapy compared with medical therapy alone (HR with CABG, 0.81; 95% CI, 0.66 to 1.00; *p* = 0.05), which became significant at 10-year follow-up [128,129].

In the REVIDED (Revascularization for Ischemic Ventricular Dysfunction) trial, PCI plus medical therapy compared to medical therapy alone failed to show that multivessel PCI improved event-free survival and LVEF among patients with severe ischemic cardiomyopathy (LVEF < 35%) and instead progressively improved with the optimization of medical therapy alone in patients with myocardium viability.

Although the REVIVED trial enrolled older patients with worse coronary disease, including left main disease, the incidences of death from any cause and the composite of death or hospitalization for heart failure were similar to the annualized rates observed in the medical therapy groups of STICH.

Nevertheless, QoL was found to be better in the PCI group up to 12 months after the difference progressively disappeared [130].

The results of REVIVED are not surprising considering the improvements in medical therapy over the past two decades, which can be appreciated when comparing the molecules and dosages used in both trials.

As a proof of this, when the REVIVED trial started (2013), angiotensin II receptor blockers (ARB); mineralocorticoid receptor antagonists (MRA); and, later on, angiotensin receptor/neprilysin inhibitors (ARNI) and sodium/glucose co-transporter-2 inhibitors (SGLT-2i) were categorized as new class I recommendations, while digitalis was downgraded to a class IIa.

Additionally, devices including cardiac resynchronization therapy (CRT) and intracardiac defibrillators (ICD) were more frequently employed in REVIVED patients than STICH patients, being a critical part of their optimal MT (OMT).

Another key difference existed in the populations enrolled: While REVIVED patients presented mainly with NYHA I and II class and were slightly symptomatic for angina, STICH patients had generally worse clinical presentation. Moreover, in the REVIVED trial, the indication for revascularization was based on myocardial viability, a criterion not considered in the STICH trial.

Bearing in mind such differences, these findings corroborate the ISCHEMIA trial results, confirming a marginal role of coronary revascularization in patients with stable CAD and optimal response to OMT.

In this specific subset of patients, surgical revascularization seems to offer prognostic advantages compared to medical therapy alone. The role of PCI remains controversial, in particular in patients with three-vessel disease.

## 5. Updates in CAD Medical Treatments

### 5.1. New Anti-Lipid Therapies

#### 5.1.1. Monoclonals Antibodies (MABS) and Long-Term Therapy

Monoclonal antibodies, which inhibit proprotein convertase subtilisin–kexin type 9 (PCSK9), are changing anti-lipid treatment, leading to reductions in cholesterol levels of up to 60%. In the FOURIER (Further Cardiovascular Outcomes Research with PCSK9 Inhibition in Subjects with Elevated Risk) trial, evolocumab compared to placebo in patients who were receiving statin therapy significantly reduced CV death, MI, stroke, hospitalization for UA, or coronary revascularization at 26-month follow-up (HR, 0.85; 95% CI, 0.79 to 0.92; *p* < 0.001) [131]. Similar results were reported with alirocumab in the ODYSSEY (Evaluation of Cardiovascular Outcomes After an Acute Coronary Syndrome During Treatment With Alirocumab) trial [132].

However, the optimal timing to initiate such treatments is still under investigation. The current use of monoclonal therapy is approved after 4–6 weeks of the maximum tolerated dosages of high-intensity statin and ezetimibe when the LDL-c target is not reached.

Lipid-lowering therapies, particularly with statins, have shown long-term reductions in lipid plaque content and stabilization of vulnerable atherosclerotic plaque. In the meta-analysis by Han-lu Lv et al., statin therapy significantly reduces all-cause mortality, CV mortality, and major coronary events over the entire follow-up period of more than 6 years. Moreover, earlier treatment with statin for 3.3–6.0 years provided an ongoing reduction in all-cause or CV for an additional 2 years [133].

PCSK9 long-term therapy proved to be effective, safe, and well tolerated without increasing the incidence of adverse events including diabetes, neurocognitive effects, and vitamin deficiency [134].

The 5-year follow-up of the FOURIER Open-Label Extension (FOURIER-OLE) showed that evolocumab reduced the rate of MACE both in patients with and without MVD. However, in patients with MVD, the benefit emerged earlier and became even higher in the later years [135].

#### 5.1.2. Inclisiran

Inclisiran is a novel approach based on silencing PCSK9 gene expression. It is a double-stranded small interfering RNA therapeutic agent that suppresses PCSK9 translation in the liver and lowers circulating concentrations of PCSK9 and LDL-C.

In patients with atherosclerotic cardiovascular disease (ASCVD) or equivalent risk of ASCVD and high LDL-c levels treated with maximum tolerated statin dose and other lipid-lowering agents, ORION 10 and 11 studies showed the safety and efficacy of Inclisiran’s administration. In both studies, inclisiran significantly reduced LDL-C levels (52% and 50% respectively; *p* < 0.001 compared to placebo) and PCSK9 levels (70% in the ORION-10 trial and 63% in the ORION-11 trial; *p* < 0.001 compared to placebo) [136].

Similarly, in the ORION-9 trial, a LDL-c levels reduction was showed in patients with heterozygous familial hypercholesterolemia and LDL-C levels ≥ 100 mg/dL despite the maximum tolerated dose of lipid-lowering therapies [137].

In a recent meta-analysis inclisiran also significantly decreased total cholesterol, ApoB, and non- HDL-C, respectively by 37%, 41%, and 45% [138].

Based on current evidence, inclisiran impact on LDL-C appears similar to that of monoclonal antibodies against PCSK9. However, we need dedicated RCTs to investigate its efficacy and safety in different clinical settings, its impact on MACE, and overall prognosis along with the persistence of LDL-C reduction with longer treatment duration [139].

### 5.2. Antithrombotic Therapy

Beyond the historical oral antiplatelet molecules, including Aspirin and the P2Y_12_ receptor inhibitors (i.e, clopidogrel, ticagrelor, and prasugrel), the recent advent of the intravenous drug cangrelor has facilitated the inhibition of platelets aggregation in the emergency context [140,141,142].

This molecule, which inhibits ADP receptor P2Y_12_, presents a rapid onset of action and a reversible platelet inhibition (plasma half-life of 3 to 6 min with complete platelet function normalization within 30 to 60 min after discontinuation).

In ACS, its administration 30 min before PCI or during the procedure does not reduce death, MI, or ischemia-driven revascularization at 48 h compared to clopidogrel [143,144,145]. Nevertheless, cangrelor significantly reduces the rate of ischemic events, including stent thrombosis during PCI without a significant increase in severe bleeding [144].

Thanks to its safety profile, cangrelor may be considered on a case-by-case basis in P2Y_12_-receptor-inhibitor-naïve ACS patients undergoing PCI when oral administration is not possible.

The availability of several antiplatelet drugs creates different opportunities to combine such molecules when double antiplatelet therapy (DAPT) is needed.

Generally, DAPT with clopidogrel is reserved after elective PCI regardless of the type of stent [46].

Differently, ACS patients—according to ESC guidelines—require a default DAPT regimen consisting of a potent P2Y_12_ receptor inhibitor and aspirin, generally recommended for 12 months [46].

However, DAPT duration should be adjusted on the individual patient’s bleeding and ischemic risk using appropriate risk scores, such as the DAPT and the PRECISE-DAPT score [146,147].

**Figure 3:**
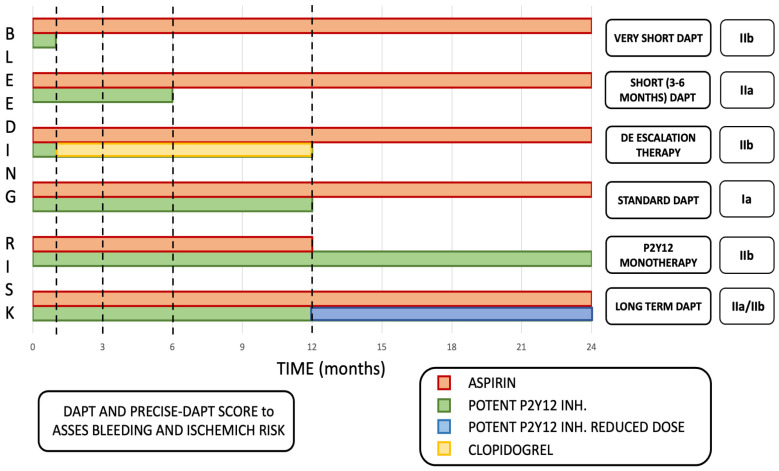
DAPT strategy in ACS patients to balance ischemic and bleeding risk [46]. Abbreviation: DAPT, dual antiplatelet therapy.

In patients at high bleeding risk, DAPT can be shortened (<6 or <12 months) by early withdrawal of the P2Y_12_-inhibitor [148,149,150,151,152,153,154]. On the contrary, in patients at high ischemic risk and without increased risk of major bleeding, DAPT can be extended (>12 months) [155,156] (Figure 3).

### 5.3. Anticoagulant Therapy Before during E Post PCI

According to ESC guidelines, in ACS patients, parenteral anticoagulation is recommended at the moment of the diagnosis, and it should be discontinued immediately after PCI. Although during PPCI the default strategy provides for the administration of bolus of unfractioned heparin (UFH), enoxaparin and bivalirudin represent a valid alternative in STEMI patients [157,158]. Differently, in NSTEMI patients who do not undergo early invasive angiography, fondaparinux is preferred to enoxaparin [159].

After revascularization, specific cases—such as thrombus in left ventricular, atrial fibrillation (AF), mechanical prosthetic valves, or mitral stenosis—require chronic anticoagulant therapy [46].

For these patients, the duration and combination of antithrombotic drugs become crucial to optimizing the ischemic and bleeding balance. In this regard, the best antithrombotic strategy has been widely investigated in patients with non-valvular AF and recent implanted stents (ACS and CCS).

In the Randomized Evaluation of Dual Antithrombotic Therapy with Dabigatran versus Triple Therapy with Warfarin in Patients with Nonvalvular Atrial Fibrillation Undergoing Percutaneous Coronary Intervention (RE-DUAL PCI) trial, dual antithrombotic therapy (DAT) with dabigatran and a P2Y_12_ inhibitor significantly reduced the rates of bleeding complications with similar ischemic event rates compared to triple antithrombotic therapy (TAT) with warfarin, a P2Y_12_ inhibitor, and aspirin [160].

Rivaroxaban, apixaban, and edoxaban showed similar results in terms of bleeding events if compared to vitamin K antagonist (VKA) administration [161,162,163].

Currently, DAT with a new oral anti-coagulant (NOAC) is recommended as the default strategy up to 12 months, after 1 week of TAT (NOAC plus DAPT consisting of aspirin and clopidogrel). However, DAT may be shortened to 6 months by withdrawing the antiplatelet therapy in patients with high bleeding risk. Differently, in patients with high ischemic risk or other anatomical/procedural characteristics that outweigh the bleeding risk, TAT should be prolonged up to 1 month, followed by DAT for up to 12 months [46].

Few data are available regarding DAT/TAT with prasugrel or ticagrelor, and today, the safety and efficacy of such combinations are not sufficiently documented.

### 5.4. Anti-Inflammatory Drugs: Colchicine Will Have a Key Role in the Therapy of the Future?

Inflammation is strictly related to the pathogenesis of atherosclerosis, and its inhibition may reduce adverse events after MI, improve the prognosis of patients with CAD, and avoid new potentially fatal major CV events.

Anti-inflammatory drugs may have a key role in slowing down and stabilizing atherosclerotic plaques, modifying the inflammatory burden. Moreover, the low cost, oral administration, and clinical CV benefits identified in patients treated with anti-inflammatory molecules for other reasons have made these drugs much attractive.

Colchicine, especially for its safety and efficacy in primary and secondary prevention in pericarditis, is considered a promising drug in acute and chronic CAD.

In the COLCOT (in the Colchicine Cardiovascular Outcomes) trial, medical therapy with low-dose colchicine (0.5 mg once daily) in patients with recent MI (<30 days) that had undergone PCI and intensive statin treatment reduced the rates of the primary end point (a composite of death from CV, resuscitated cardiac arrest, MI, stroke, or urgent hospitalization) compared to the placebo group (HR, 0.77; 95% CI, 0.61 to 0.96; *p* = 0.02) [164].

Regarding CCS, in the Low-dose Colchicine trial-2 (LoDoCo2) trial, patients who received colchicine (0,5 mg once daily) presented fewer CV adverse events compared to placebo (HR, 0.69; 95% CI, 0.57 to 0.83; *p* < 0.001) [165].

However, the optimal timing to administrate colchicine is not well established, while some studies recommend starting the treatment within 3 days after MI.

As atherosclerosis is a continuous inflammatory disorder within the arterial wall, long-term anti-inflammatory therapy has the potential to improve the prognosis of patients with CAD. Long-term treatment with colchicine seems to reduce the risk of MACE, reducing plaque instability, particularly in low-intensity plaque volumes. Indeed, in a meta-analysis by Xia et al. colchicine treatment reduced significantly the incidence of MACE after 6- and 12-month follow-up [166]. Moreover, after 1-year follow-up, anti-inflammatory treatment reduced the rates of MI, stroke, and revascularization without having a significant effect on all-cause mortality, CV, or non-CV mortality [167]. Long term treatment with colchicine increases gastrointestinal adverse effects, which might be partially mitigated by the administration of lower doses without nullifying the CV benefits. Importantly, long-term therapy does not increase the risk of sepsis, cancer, cytopenia, and myotoxicity [168].

### 5.5. Sodium–Glucose Co-Transporter 2 Inhibitors (SGLT2i)

Pharmacological inhibition of SGLT2 reduces urinary glucose reuptake, improving glycemia control, and reducing weight and blood pressure. Additionally, SGLT2i may slow the development of atheroma, reducing inflammation through cytokine inhibition (e.g., monocyte chemoattractant protein (MCP)-1, interleukin (IL)-6, IL-10, tumor necrosis factor (TNF)-α and IL-1β) and improving endothelial function and oxidative stress. However, there is better control of dyslipidemia with lower serum total cholesterol and triglyceride levels, secondarily impacting atheroma growth. Furthermore, SGLT2i reduces leukocyte adhesion to endothelial cells and their transmigration into the intra-intimal space by inhibiting the endothelial adhesion molecules such as the vascular cell adhesion molecule (VCAM)-1 and the intracellular adhesion molecule (ICAM)-1. These mechanisms, associated with reduced macrophage infiltration and formation of cholesterol esters, improve the plaque composition and plaque stability [169,170,171].

SGLT2i have a cardioprotective effect reducing CV mortality, all-cause mortality, and new hospitalization for heart failure, especially in patients with reduced LVEF with or without diabetes [172].

In the Empagliflozin in acute myocardial infarction (EMMY) trial, patients treated with 10 mg empaglifozin once daily showed significantly improved LVEF compared to the placebo group (*p* = 0.029) as well as NT-proBNP value, left-ventricular end-systolic, and end-diastolic volumes after 6-month follow-up [173].

Despite limited evidence, in patients with HF with preserved LVEF, dapagliflozin and empagliflozin have been shown to significantly reduce the risk of CV death and hospitalization for HF regardless of the presence of type 2 diabetes [174,175].

These initial results are encouraging and some ongoing RCTs with larger sample size will try to confirm dapagliflozin and empagliflozin’s clinical benefits in this context.

## 6. CAD and Coronavirus Disease 2019 (COVID-19)

In previous clinical studies, a strict interplay between COVID-19 and CV diseases has been observed. Although the predominant clinical manifestation of COVID-19 is viral pneumonia, it has also been related to CV disorders such as MI, arrhythmias, and thromboembolism. On the other hand, patients with CV disorders, especially CAD, were shown to be more prone to severe progression and multiple complications of COVID-19.

The data from the Kermani–Alghoraishi review suggest that COVID-19, especially the most severe forms, may play a role in the progression and development of ACS, especially STEMI, due to increased thrombogenicity, as seen during coronary angiography in this subset of patients [176].

The mechanisms underlying COVID-19-induced ACS might involve plaque rupture, coronary spasm, or microthrombi, owing to systemic inflammation and cytokine storm [177,178]. A direct endothelial or vascular injury caused by the infection might also increase the risk of thrombus formation and therefore ACS [179].

Despite the higher prevalence of coagulation abnormalities in patients with COVID-19, the risk–benefit profile favors PPCI over thrombolysis [180]. Currently, there is insufficient information to standardize percutaneous treatment, but in the context of this amplified thrombotic burden, prolonged and accurate thrombospiration combined with more aggressive intraprocedural antithrombotic therapy may be beneficial. Adequate plaque assessment is crucial for determining the optimal revascularization strategy. Although no randomized controlled trials (RCTs) are available today, it is reasonable to believe that minimizing the implantation of metallic materials could be advantageous due to the increased risk of intrastent thrombosis in these patients. Additionally, the post-PCI medical treatment approach must consider the higher ischemic risk to optimize the dual antiplatelet therapy (DAPT) strategy.

## 7. New Devices and Procedural Advancements for PCI and CABG

### 7.1. Newer Technology: Drug Coated Balloons (DCBs), Bioresorbable Vascular Scaffolds (BVS), and DynamX Bioadaptor

New generation DES are the most common devices used in modern PCI, improving CV outcomes compared to POBA, older DES, or BMS. Despite the great results obtained in terms of safety and efficacy, new technologies have been introduced in clinical practice in recent years.

Drug-coating balloon (DCB) technology is based on the delivery of the antiproliferative drugs to the vessel wall without leaving any permanent or semipermanent prosthesis, thus reducing the risk of stent failure, the need for long-term DAPT and associated bleeding complications, and maintaining the coronary vasomotor response and vessel geometry with proven positive remodeling. Moreover, DCBs do not exclude the possibility of future surgical treatments. At the moment, their efficacy and safety have been well demonstrated in the setting of in-stent restenosis (ISR) and small-vessel disease [181,182,183,184,185,186,187,188].

However, the use of DCB technology might offer advantages in ACS patients while restoring coronary flow and promoting vascular healing without the risks connected to inadequate stent sizing. In the REVELATION (Drug-Coated Balloon Versus Drug-Eluting Stent in Acute Myocardial Infarction) trial for ST-segment elevation myocardial infarction, no difference was observed between the DCB and DES groups, even at two-year follow-up in terms of MACE [189].

Bioresorbable vascular scaffolds (BVS) were designed to improve late event-free survival with similar mechanical support of DES but with complete resorption leading the vascular structure and function to recover. However, studies have demonstrated that BVS are inferior to contemporary DES throughout the first 5 years, increasing patients’ risk of serious adverse CV events and the need for reintervention of the target lesion during this time [190].

The new DynamX Bioadaptor uses a drug-eluting bioresorbable polymer coating that dissolves after three months. It is designed to restore coronary artery hemodynamic normality by restored vessel pulsatility, compliance, adaptive increase in blood flow volume as well as to provide plaque stabilization and regression. The first RCT showed non-inferiority on target lesion failure rate after 12-month of follow-up, with lower late lumen loss and improved vessel function compared with standard DES [191].

### 7.2. Robotic PCI

Robotic-assisted PCI is one of the novelties within interventional cardiology, aiming to reduce both radiation exposure and orthopedic injuries for operators, while maintaining procedural quality and safety.

The available systems are made up of two subunits—the robotic arm that is placed bedside, and the interventional cockpit, where the primary operator sits to perform the PCI.

State of the art, multicenter studies have shown a significant reduction in median radiation exposure without increasing CV events at 30-day follow-up [192].

These results are encouraging, and some RCTs with larger sample sizes will be necessary to better understand the real impact of this technology.

### 7.3. On-Pump and Off-Pump CABG, Minimalist Approach

Off-pump CABG consists in a surgical technique avoiding the use of extracorporeal circulation with obvious benefits particularly in high-risk patients with extensive aortic atherosclerotic disease, renal impairment, cerebrovascular disease, and bleeding dyscrasias.

However, in the Randomized On/Off Cardiopulmonary Bypass (ROOBY) trial, off-pump CABG did not lead to advantages in terms of 10-year death or revascularization end points compared to the traditional on-pump strategy [193].

Totally endoscopic coronary artery bypass is a less invasive technique, reducing hospitalization times [194,195]. However, this is limited to internal thoracic artery (ITA) bypass. This strategy can be appealing, particularly in patients with single-vessel disease who are sent to surgery or when a hybrid coronary revascularization with PCI is considered.

## 8. Conclusions

The acute and the chronic coronary syndromes have been extensively studied over the past decades to optimize the diagnostic process, the revascularization strategy and the medical treatment.

The attempt to reduce the invasiveness and the timescales makes CT increasingly important among other tests in the diagnostic algorithm. Moreover, the possibility to implement anatomical data with functional assessments makes CT very attractive in evaluating vessel-specific ischemia and in guiding decision-making regardless of the use of coronary angiography.

In ACS patients, invasive revascularization (PCI or CABG) is the gold standard. On the contrary, considering the progresses of pharmaceutic in this field, optimal medical therapy remains the landmark of treatment and the first choice in the majority of stable CAD, where future directions are targeting the inflammatory burden to stop or slow down the atherosclerosis process.

## Figures and Tables

**Table 1:** Functional testing in suspected CAD.

Functional Testing				
	SEN	SPE	Advantages	Limitations
EXERCISE ECG	54%	58%	Non-invasive No radiation Widely available and low cost Functional assessment	No value in presence of LBBB, WPW syndrome, paced rhythm, treatment with digitalis, ST depression at resting time Operator and patient dependance No ischemia segment localization
ECHO STRESS	76%	80%	Non-invasive No radiation Structural heart information Ischemia segment localization	Operator and patient dependance Lower sensitivity for single vessel disease Imaging windows dependance
CMR STRESS	84%	85%	No radiation Subendocardial perfusion assessment Viability assessment Structural heart information	Higher cost Unable in GFR < 30 mL/min/1.73 m^2^ Less available Claustrophobia
PET STRESS	85%	86%	Absolute quantification of perfusion defect	Higher cost Radiation exposure Less available
SPECT	81%	78%	Relative quantification of perfusion defect Quantitative assessment possible	Radiation exposure Patient’s limitations

Abbreviations: SPECT, single-photon-emission computed tomography; PET, positron emission tomography; CMR, cardiac magnetic resonance; LBBB, left bundle branch block; WPW syndrome, Wolff–Parkinson–White syndrome; GFR, glomerular filtration rate; SEN, sensitivity; SPE, specificity.

**Table 2:** Anatomical testing in suspected CAD.

Anatomical Testing				
	SEN	SPE	Advantages	Limitations
CCTA	96%	79%	Non-invasive Widely available Obstructive CAD assessment Structural heart information Functional assessment (CT-FFR)	Radiation exposure Unable to confirm ischemia Motion artefacts Calcification overestimation
CACS	58%	62%	Non-invasive Widely available No contrast used	No lumen stenosis information Radiation exposure (modest dose)
ICA	100%	100%	Gold standard Revascularization during the same procedure Functional assessment (FFR, IFR)	Radiation exposure Procedural risk complications Trained staff Use of contrast Unable to confirm ischemia

Abbreviations: CCTA, coronary computed tomography angiography; CACS, coronary artery calcium score; CT-FFR, computed tomography–fractional flow reserve; CAD, coronary artery disease; FFR, fractional flow reserve; IFR, instantaneous flow reserve; SEN, sensitivity; SPE, specificity.

**Table 3:** Highlighted indications for cardiovascular computed tomography in patients with acute and stable chest pain [45].

COR	LOE	Indications for CCT
		Acute Chest Pain
1	A	For intermediate-risk patients with acute chest pain and no known CAD eligible for diagnostic testing after a negative or inconclusive evaluation for ACS, CCTA is useful for exclusion of atherosclerotic plaque and obstructive CAD.
2a	B-NR	For intermediate-risk patients with acute chest pain and known nonobstructive CAD, CCTA can be useful to determine progression of atherosclerotic plaque and obstructive CAD.
		Stable Chest Pain
1	A	For intermediate-high risk patients with stable chest pain and no known CAD, CCTA is effective for diagnosis of CAD, for risk stratification, and for guiding treatment decisions.
2a	B-R	For patients with stable chest pain and no known CAD categorized as low risk, CAC testing is reasonable as a first-line test for excluding calcified plaque and identifying patients with a low likelihood of obstructive CAD.
2a	B-NR	For intermediate–high risk patients with stable chest pain and no known CAD undergoing stress testing, the addition of CAC testing can be useful.
2a	B-NR	For patients who have stable chest pain with previous coronary revascularization, CCTA is reasonable to evaluate bypass graft or stent patency (for stents ≥ 3 mm).
2a	B-NR	For symptomatic patients with known nonobstructive CAD who have stable chest pain, CCTA is reasonable for determining atherosclerotic plaque burden and progression to obstructive CAD and guiding therapeutic decision-making.

Abbreviations: COR, class of recommendation; LOE, level of evidence; CCT, cardiovascular computed tomography; CCTA, coronary computed tomography angiography; CAC, coronary artery calcium.
